# Large vessel occlusion stroke outcomes in diabetic vs. non-diabetic patients with acute stress hyperglycemia

**DOI:** 10.3389/fnins.2023.1073924

**Published:** 2023-01-27

**Authors:** Honglian Duan, Ho Jun Yun, Gary Benjamin Rajah, Fengli Che, Yanling Wang, Jing Liu, Yanna Tong, Zhe Cheng, Lipeng Cai, Xiaokun Geng, Yuchuan Ding

**Affiliations:** ^1^Department of Neurology, Beijing Luhe Hospital, Capital Medical University, Beijing, China; ^2^Department of Neurosurgery, School of Medicine, Wayne State University, Detroit, MI, United States; ^3^Department of Neurosurgery, Munson Healthcare, Munson Medical Center, Traverse City, MI, United States

**Keywords:** stress-induced hyperglycemia, GAR index, premorbid diabetic status, large vessel occlusion stroke, outcome

## Abstract

**Objective:**

This study assesses whether stress-induced hyperglycemia is a predictor of poor outcome at 3 months for patients with acute ischemic stroke (AIS) treated by endovascular treatment (EVT) and impacted by their previous blood glucose status.

**Methods:**

This retrospective study collected data from 576 patients with AIS due to large vessel occlusion (LVO) treated by EVT from March 2019 to June 2022. The sample was composed of 230 and 346 patients with and without diabetes mellitus (DM), respectively, based on their premorbid diabetic status. Prognosis was assessed with modified Rankin Scale (mRS) at 3-month after AIS. Poor prognosis was defined as mRS>2. Stress-induced hyperglycemia was assessed by fasting glucose-to-glycated hemoglobin ratio (GAR). Each group was stratified into four groups by quartiles of GAR (Q1–Q4). Binary logistic regression analysis was used to identify relationship between different GAR quartiles and clinical outcome after EVT.

**Results:**

In DM group, a poor prognosis was seen in 122 (53%) patients and GAR level was 1.27 ± 0.44. These variables were higher than non-DM group and the differences were statistically significant (*p* < 0.05, respectively). Patients with severe stress-induced hyperglycemia demonstrated greater incidence of 3-month poor prognosis (DM: Q1, 39.7%; Q2, 45.6%; Q3, 58.6%; Q4, 68.4%; *p* = 0.009. Non-DM: Q1, 31%; Q2, 32.6%; Q3, 42.5%; Q4, 64%; *p* < 0.001). However, the highest quartile of GAR was independently associated with poor prognosis at 3 months (OR 3.39, 95% CI 1.66–6.96, *p* = 0.001), compared to the lowest quartile in non-DM patients after logistic regression. This association was not observed from DM patients.

**Conclusion:**

The outcome of patients with acute LVO stroke treated with EVT appears to be influenced by premorbid diabetes status. However, the poor prognosis at 3-month in patients with DM is not independently correlated with stress-induced hyperglycemia. This could be due to the long-term damage of persistent hyperglycemia and diabetic patients’ adaptive response to stress following acute ischemic damage to the brain.

## Introduction

Approximately one-third of patients with acute ischemic stroke (AIS) develop hyperglycemia regardless of history of diabetes (Li et al., 2013; Yao et al., 2016; Almobarak et al., 2020; Jiang et al., 2021). The acute stress response of the hypothalamic-pituitary-adrenal axis and the sympathetic nervous system in response to brain injuries are believed to be responsible (Christensen et al., 2004). Hyperglycemia is associated with increased hemorrhagic transformation and can be detrimental to patients with AIS (Robbins and Swanson, 2014; Rosso et al., 2018; Suissa et al., 2020). Larger ischemic areas, worse functional, and cognitive outcomes, and increased mortalities are found to be independently linked to increased blood glucose levels at the time of hospital admission (Tsivgoulis et al., 2019). Stress-induced hyperglycemia increases the risk of stroke recurrence and overall mortality in AIS patients even without history of diabetes (Zhu et al., 2019).

Patients without premorbid diabetes mellitus (DM) tend to develop other morbidities when subjected to stress-induced hyperglycemia than those with history of DM. SITS study notes potential disparities of early mortality and symptomatic intracranial hemorrhage (sICH) in association of hyperglycemia and history of DM; in patients without history of diabetes, admission hyperglycemia was linked to a higher rate of sICH and mortality as well as worse functional independence at 3 months (Ahmed et al., 2010). Similar results have been found in AIS patients who received intravenous thrombolysis (IVT); risk of death, hemorrhagic complications, and functional dependency drastically increase with severe stress-induced hyperglycemia in those without history of DM (Merlino et al., 2022).

High glucose levels have been considered as a predictor of poor functional outcome after IVT and mechanical thrombectomy (MT) (Chen et al., 2019; Tsivgoulis et al., 2019; Suissa et al., 2020; Merlino et al., 2021a; Shen et al., 2021; Cannarsa et al., 2022). Merlino et al. (2022) have found that risk of functional dependence, mortality, and hemorrhagic complications was increased in patients with more severe stress hyperglycemia after IVT only in the absence of prior diabetic history. It is not clear whether the same results would occur to those after MT instead of IVT. In another study, the same research group has concluded that patients with AIS and severe stress hyperglycemia tend to have poor outcome at 3 months after undergoing MT. However, whether the poor outcome was due to premorbid diabetes mellitus (DM) or stress hyperglycemia remains largely unknown (Merlino et al., 2021a). One available study notes that the predictive power of stress-induced hyperglycemia ratio (>0.96) for adverse outcome is not significant in diabetic patients. However, this finding is based on a small sample size (*n* = 39) (Chen et al., 2019). Overall, there is an insufficient number of studies to determine an association between hyperglycemia and clinical outcomes after MT.

This study was designed to assess whether stress-induced hyperglycemia could function as a predictor of AIS patients’ poor outcomes who had undergone emergent endovascular treatment (EVT). Large samples were obtained to definitively address differences in stress-induced hyperglycemia and prognosis between DM and non-DM patients with AIS.

## Materials and methods

### Study population

A retrospective observational study was performed by collecting data from patients with AIS due to large vessel occlusion (LVO) from March 2019 to June 2022. The patients underwent EVT (mechanical thrombectomy and/or stent implantation) in Beijing Luhe Hospital, Capital Medical University, within 24 h from the onset of symptom(s). Each patient was assessed by an experienced neurologist. The inclusion criteria for enrollment were: (1) Age ≥ 18 years, (2) diagnosis of AIS based on the definition established by the World Health Organization, and (3) EVT. The exclusion criteria were: (1) no data on fasting plasma glucose or HbA1C and (2) lost to follow up.

### Data collection and clinical assessment

Diagnostic studies were obtained, including neurologic examinations, blood tests, echocardiography, electrocardiograph, and appropriate brain imaging studies. Relevant past medical histories were acquired, such as hypertension, DM, coronary heart disease, atrial fibrillation, smoking, hyperlipemia, and past ischemic stroke. Fasting plasma specimens were collected within 24 h from the onset of symptoms. Diabetic patients were defined as those with a history of diabetes mellitus or HbA1c ≥ 6.5%. The stroke subtypes were confirmed by 2 neurologists according to TOAST. Stroke severity was assessed on admission and at discharge by certified examiners, using the National Institutes of Health Stroke Scale (NIHSS). All patients were assessed on 3 months, using modified Rankin Scale (mRS) and poor prognosis was defined as mRS>2. Mortality within 7 and 30 days and presence of sICH or intracranial hemorrhage (ICH) were checked as part of the routine clinical practice.

### Assessment of stress hyperglycemia

There is no standardized diagnostic criteria for acute stress hyperglycemia. Random blood glucose or fasting blood glucose levels are generally measured when analyzing stress-induced hyperglycemia after AIS. However, these serum studies are often influenced by eating and/or previous glucose status.

Fasting glucose levels were measured after admission to diagnose “absolute” stress hyperglycemia since most of the blood samples were collected from 12 to 24 h after AIS. Fasting plasma glucose (mmol/l) to HbA1c ratio (GAR) was measured to diagnose “relative” hyperglycemia since GAR minimizes the impact of diet and previous blood glucose status and was associated with critical illness (Roberts et al., 2015, 2021; Su et al., 2017; Li et al., 2020). The diabetic and non-diabetic groups were divided by applying quartiles of GAR. The quartiles were used to assess whether higher GAR was associated with worse prognosis in these two groups. GAR quartiles were applied to divide the patients into four groups (Q1–Q4).

### Statistical analysis

Statistic package for social science (SPSS) 19.0 software was used to analyze the data. Categorical variables were presented in frequencies and percentages from chi-square test or Fisher’s exact test. Continuous variables were expressed as means with standard deviations and statistical analyses were performed using one-way ANOVA test and *t*-test. Binary logistic regression analysis was used to assess the effect of stress hyperglycemia, using the lowest GAR index as a reference and comparing prognosis. The regression model was adjusted for all potential confounders. The probability value for univariate analysis was less than 0.1. The specific variables for the adjustments considered in each analysis were indicated in Table 1. *p*-value < 0.05 was considered statistically significant.

**TABLE 1:** GAR and 3-month prognosis between DM patients and non-DM patients.

	DM (*n* = 230)	Non-DM (*n* = 346)	*P*
GAR	1.27 (0.44)	1.18 (0.38)	<0.001
mRS on 90 days mRS > 2), n (%)	122 (53)	147 (42.5)	0.014

DM, Diabetes mellitus; GAR, glucose-to-glycated hemoglobin ratio; mRS, modified Rankin Score.

## Results

### Baseline characteristics

**FIGURE 1:**
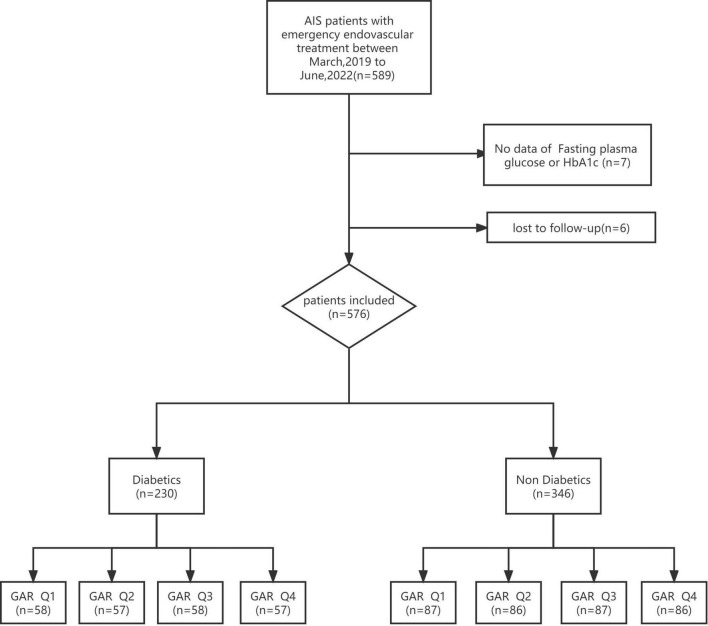
Flowchart of study participants. AFIS, acute ischemic stroke; HbA1c, glycated hemoglobin; GAR Q1, first glucose-to-glycated hemoglobin ratio quartiles; GAR Q2, second glucose-to-glycated hemoglobin ratio quartiles; GAR Q3, third glucose-to-glycated hemoglobin ratio quartiles; GAR Q4, fourth glucose-to-glycated hemoglobin ratio quartiles.

A total of 589 patients were treated by EVT. Seven patients without fasting glucose or HbA1c values and six patients who were lost to follow-up were excluded. The remaining patients (*n* = 576) were divided into DM (*n* = 230) and non-DM (*n* = 376) group. The two groups were then divided into 4 quartiles according to GAR (Figure 1).

**FIGURE 2:**
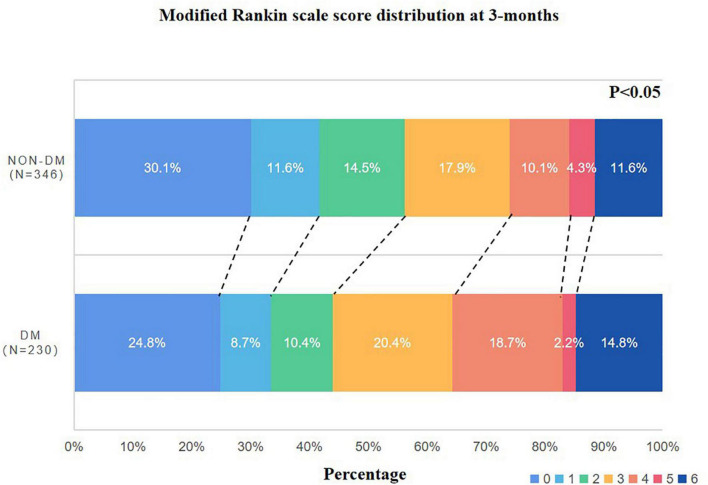
Distribution of mRS scores at 3 months between patients with and without DM.

In the DM group, poor outcomes were found in 122 patients (53%) and the GAR level was 1.27 ± 0.44. These two parameters were higher than the non-DM group (*p* < 0.05, respectively) (Table 2). The mRS distribution was different between the two groups; more patients with DM had higher mRS scores (Figure 2).

**TABLE 2:** Demographics, clinical data, complications and functional outcomes of non-diabetic patients.

	GAR Q1 (*n* = 87)	GAR Q2 (*n* = 86)	GAR Q3 (*n* = 87)	GAR Q4 (*n* = 86)	*P*
Age, years	65.18 ± 12.33	66.01 ± 12.80	67.15 ± 11.16	67.41 ± 13.62	0.621
Male, n (%)	59 (67.8)	57 (66.3)	53 (60.9)	38 (44.2)	0.006
**Vascular risk factors**					
Hypertension, n (%)	49 (56.3)	54 (62.8)	43 (49.4)	46 (53.5)	0.346
Coronary heart disease, n (%)	9 (10.3)	14 (16.3)	17 (19.5)	16 (18.6)	0.350
Atrial fbrillation, n (%)	12 (13.8)	14 (16.3)	19 (21.8)	18 (20.9)	0.466
Previous stroke, n (%)	13 (14.9)	15 (17.4)	19 (21.8)	14 (16.3)	0.658
Current smoking, n (%)	45 (51.7)	40 (46.5)	32 (36.8)	23 (26.7)	0.004
Hyperlipidemia, n (%)	12 (13.8)	11 (12.8)	18 (20.7)	12 (14.0)	0.451
**Laboratory findings**					
Fasting plasma glucose (mmol/l)	4.92 ± 0.44	5.79 ± 0.38	6.83 ± 0.65	9.56 ± 2.35	<0.001
HbA1c values (%)	5.79 ± 0.28	5.79 ± 0.31	5.75 ± 0.34	5.73 ± 0.34	0.386
GAR	0.85 ± 0.06	0.99 ± 0.04	1.19 ± 0.07	1.68 ± 0.43	<0.001
Total bilirubin (mmol/l)	17.38 ± 13.38	18.45 ± 11.54	18.58 ± 8.15	15.59 ± 7.84	0.227
Direct bilirubin (mmol/l)	5.28 ± 2.51	5.85 ± 3.07	6.21 ± 3.40	5.29 ± 2.69	0.116
Triglyceride (mmol/l)	1.30 ± 0.83	1.13 ± 0.59	1.04 ± 0.66	1.19 ± 1.0	0.175
Total cholesterol (mmol/l)	4.29 ± 0.97	4.18 ± 1.06	4.00 ± 0.99	4.24 ± 1.17	0.302
High-density lipoprotein (mmol/l)	1.12 ± 0.31	1.09 ± 0.25	1.34 ± 1.41	1.25 ± 0.37	0.126
Low-density lipoprotein (mmol/l)	2.79 ± 0.82	2.72 ± 0.87	2.49 ± 0.84	2.62 ± 0.94	0.124
Homocysteine (umol/l)	20.46 ± 23.21	20.54 ± 27.95	17.53 ± 15.02	15.17 ± 10.11	0.246
C-reactive protein (mg/l)	15.61 ± 19.83	14.35 ± 20.68	23.17 ± 41.88	22.44 ± 38.29	0.167
**Treatment emergency**					
Intravenous thrombolysis, n (%)	29 (33.3)	25 (29.1)	19 (21.8)	24 (27.9)	0.405
Lesion of anterior circulation, n (%)	16 (18.4)	10 (11.6)	20 (23.0)	15 (17.4)	0.274
Stroke subtypes (TOAST classification)					0.006
LAA, n (%)	58 (66.7)	43 (50)	41 (47.1)	38 (44.2)	
CE, n (%)	16 (18.4)	32 (37.2)	25 (28.7)	23 (26.7)	
Other, n (%)	13 (14.9)	11 (12.8)	21 (24.1)	25 (29.1)	
**NIHSS**					
At admission	14.24 ± 5.49	14.55 ± 5.64	16.05 ± 6.05	16.38 ± 5.23	0.026
At discharge	6.05 ± 7.69	6.93 ± 9.16	9.89 ± 11.28	14.37 ± 12.58	<0.001
**Complication**					
Any ICH, n (%)	31 (35.6)	34 (39.5)	37 (42.5)	47 (54.7)	0.068
SICH, n (%)	3 (3.4)	10 (11.6)	10 (11.5)	20 (23.3)	0.001
Malignant edema, n (%)	14 (16.1)	18 (20.9)	30 (34.5)	39 (45.3)	<0.001
Respiratroy failure, n (%)	7 (8.0)	5 (5.8)	5 (5.7)	13 (15.1)	0.095
**Mortality**					
Within 7 days, n (%)	3 (2.4)	3 (3.5)	2 (2.3)	13 (15.1)	0.001
Within 30 days, n (%)	4 (4.6)	7 (8.1)	12 (13.8)	22 (25.6)	<0.001
mRS on 3 months (mRS > 2), n (%)	27 (31)	28 (32.6)	37 (42.5)	55 (64)	<0.001

Data were expressed as mean+standard deviation (SD), number (percent).

GAR, glucose-to-glycated hemoglobin ratio; GAR Q1 ≤ 0.93, first GAR quartile; 0.93 < GAR Q2 ≤ 1.075, second GAR quartile; 1.075 < GAR Q3 ≤ 1.331, third GAR quartile; GAR Q4 > 1.331, fourth GAR quartile.LAA, Large -artery atherosclerosis; CE, Cardioembolism; Other, stroke of other determined etiology & Stroke of other undetermined etiology; NIHSS, the National Institutes of Health Stroke Scale; ICH, intracranial hemorrhage; SICH, symptomatic intracranial hemorrhage; mRS, modified Rankin score.

**FIGURE 3:**
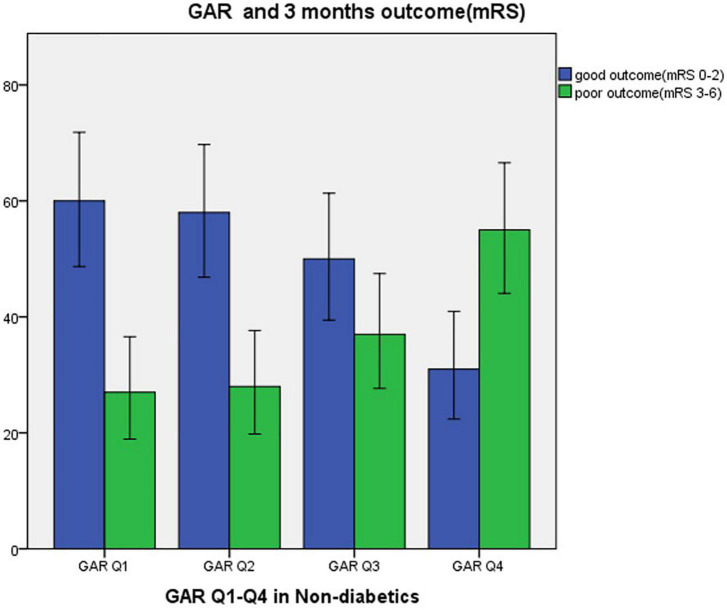
Three months outcome by GAR quartile in non-diabetic patients. GAR Q1, first glucose-to-glycated hemoglobin ratio quartile; GAR Q2, second glucose-to-glycated hemoglobin ratio quartile; GAR Q3, third glucose-to-glycated hemoglobin ratio quartile; GAR Q4, fourth glucose-to-glycated hemoglobin ratio quartile.

The following numbers of GAR quartiles represented patients without DM: 87 (25.1%) in the first and third quartiles, and 86 patients (24.9%) in the second and fourth quartiles. The general characteristics of each GAR quartile were assessed (Table 3). Compared to the other three quartiles, the fourth quartile had a significantly lower rate of male gender, current smoking history, and large-artery atherosclerosis (LAA) stroke, but higher rate of fasting plasma glucose, NIHSS at admission and at discharge, sICH, malignant edema, mortality within 7 and 30 days, and poor outcome in 3-month (*p* < 0.05, respectively). Patients with more severe stress hyperglycemia had a higher prevalence of poor outcome at 3 months (GAR Q1, 31%; Q2, 32.6%; Q3, 42.5%; Q4, 64%; *p* < 0.01) (Figure 3).

**TABLE 3:** Demographics, clinical data, complications and functional outcomes of diabetic patients.

	GAR Q1 (*n* = 58)	GAR Q2 (*n* = 57)	GAR Q3 (*n* = 58)	GAR Q4 (*n* = 57)	*P*
Age, years	67.76 ± 12.24	68.61 ± 8.59	65.52 ± 10.86	70.21 ± 9.65	0.621
Male, n (%)	27 (46.6)	29 (50.9)	33 (56.9)	32 (56.1)	0.652
**Vascular risk factors**					
Hypertension, n (%)	38 (65.5)	40 (70.2)	46 (79.3)	37 (64.9)	0.302
Coronary heart disease, n (%)	22 (37.9)	16 (28.1)	15 (25.9)	16 (28.1)	0.491
Atrial fbrillation, n (%)	12 (20.7)	17 (29.8)	12 (20.7)	9 (15.8)	0.327
Previous stroke, n (%)	11 (19)	10 (17.5)	19 (32.8)	20 (35.1)	0.060
Current smoking, n (%)	17 (29.3)	16 (28.1)	19 (32.8)	18 (31.6)	0.947
Hyperlipidemia, n (%)	20 (34.5)	13 (22.8)	15 (25.9)	14 (24.6)	0.500
**Laboratory findings**					
Fasting plasma glucose (mmol/l)	6.56 ± 1.74	8.13 ± 1.59	9.92 ± 2.13	14.94 ± 3.62	<0.001
HbA1c (%)	8.17 ± 2.06	7.59 ± 1.49	7.47 ± 1.49	7.96 ± 1.33	0.386
GAR	0.81 ± 0.13	1.07 ± 0.05	1.33 ± 0.08	1.88 ± 0.38	<0.001
Total bilirubin (mmol/l)	13.98 ± 5.79	18.26 ± 8.35	14.90 ± 6.42	16.27 ± 8.91	0.017
Direct bilirubin (mmol/l)	4.51 ± 1.77	6.20 ± 3.25	4.84 ± 2.09	5.37 ± 2.91	0.004
Triglyceride (mmol/l)	1.26 ± 0.72	1.25 ± 0.55	1.36 ± 0.95	1.39 ± 0.69	0.175
Total cholesterol (mmol/l)	3.93 ± 1.03	4.24 ± 1.14	4.34 ± 1.48	4.31 ± 0.97	0.302
High-density lipoprotein (mmol/l)	1.02 ± 0.26	1.10 ± 0.25	1.15 ± 0.28	1.12 ± 0.26	0.039
Low-density lipoprotein (mmol/l)	2.53 ± 0.85	2.76 ± 0.93	2.73 ± 1.22	2.77 ± 0.77	0.124
Homocysteine (umol/l)	17.73 ± 9.89	17.52 ± 13.39	13.12 ± 5.95	16.49 ± 11.27	0.246
C-reactive protein (mg/l)	13.30 ± 17.96	18.09 ± 19.16	23.42 ± 42.52	15.78 ± 18.65	0.167
**Treatment emergency**					
Intravenous thrombolysis, n (%)	17 (29.3)	18 (31.6)	10 (17.2)	17 (29.8)	0.284
Lesion of anterior circulation, n (%)	10 (17.2)	8 (14)	15 (25.9)	12 (21.1)	0.416
Stroke subtypes (TOAST classification)					0.457
LAA, n (%)	33 (56.9)	29 (50.9)	31 (53.4)	34 (59.6)	
CE, n (%)	16 (27.6)	23 (40.4)	17 (29.3)	19 (33.3)	
Other, n (%)	9 (15.5)	5 (8.8)	10 (17.2)	4 (7.0)	
**NIHSS**					
At admission	12.52 ± 7.88	14.90 ± 5.67	15.29 ± 6.12	15.35 ± 6.35	0.037
At discharge	8.88. ± 10.28	8.04 ± 8.98	10.62 ± 11.46	14.56 ± 12.58	0.005
**Complication**					
Any ICH, n (%)	24 (41.4)	24 (42.1)	30 (51.7)	28 (49.1)	0.608
SICH, n (%)	4 (6.9)	3 (5.3)	13 (22.4)	14 (24.6)	0.003
Malignant edema, n (%)	10 (17.2)	14 (24.6)	19 (32.8)	24 (42.1)	0.026
Respiratroy failure, n (%)	4 (6.9)	3 (5.3)	8 (13.8)	7 (12.3)	0.336
**Mortality**					
Within 7 days, n (%)	3 (5.2)	2 (3.5)	7 (12.1)	9 (15.8)	0.075
Within 30 days, n (%)	4 (6.9)	4 (7.0)	12 (20.7)	16 (28.1)	0.002
mRS on 3 months (mRS > 2), n (%)	23 (39.7)	26 (45.6)	34 (58.6)	39 (68.4)	0.009

Data were expressed as mean ± standard deviation (SD), number (percent).

GAR, glucose-to-glycated hemoglobin ratio; GAR Q1 ≤ 0.986, first GAR quartile; < 0.986GAR Q2 ≤ 1.187,second GAR quartile; 1.187 < GAR Q3 ≤ 1.497, third GAR quartile; GAR Q4 > 1.497, fourth GAR quartile. LAA, Large -artery atherosclerosis; CE, Cardioembolism; Other, stroke of other determined etiology & Stroke of other undetermined etiology; NIHSS, the National Institutes of Health Stroke Scale; ICH, intracranial hemorrhage; SICH, symptomatic intracranial hemorrhage; mRS, modified Rankin score.

The following numbers of GAR quartiles represented patients with DM: 58 (25.2%) in the first and third quartiles, and 57 (24.8%) in the second and fourth quartiles (Table 4). There were no significant differences in age, sex, or vascular risk factors among the four groups. The fourth GAR quartile had higher fasting plasma glucose, total bilirubin, direct bilirubin, and NIHSS at admission and discharge. The fourth quartile had a significantly higher rate of sICH, malignant edema, mortality within 30 days, and poor outcome at 3-month. The etiology of AIS was assessed and there was no significant difference among the four quartiles.

**TABLE 4:** Outcome Complications by Logistic Regression Model [ORs (95% CIs) of outcome measures adjusted for GAR quartiles].

		Unadjusted OR (95% CI)	*P*	Adjusted OR (95% CI)	*P*
**DM group (*n* = 230)**					
30 days mortality	GAR				
	Q1	Reference		Reference	
	Q2	0.67 (0.14–3.18)	0.617	0.82 (0.16–4.34)	0.819
	Q3	2.44 (0.71–8.38)	0.155	2.72 (0.71–10.36)	0.144
	Q4	3.73 (1.18–11.77)	0.025	4.36 (1.25–15.25)	0.021
	Hypertension	0.54 (0.26–1.12)	0.100	0.48 (0.21–1.21)	0.090
	Any ICH	3.71 (1.69–8.11)	0.001	3.52 (1.48–8.38)	0.004
	Respiratory failure	9.20 (3.59–23.57)	<0.001	9.61 (3.37–27.41)	<0.001
3-month poor outcome (mRS > 2)	GAR				
	Q1	Reference		Reference	
	Q2	0.80 (0.36–1.77)	0.583	0.76 (0.31–1.86)	0.550
	Q3	1.23 (0.56–2.67)	0.607	1.11 (0.46–2.71)	0.811
	Q4	2.51 (1.19–5.30)	0.016	2.06 (0.88–4.79)	0.095
	Age	1.03 (1.00–1.05)	0.041	1.03 (0.99–1.06)	0.094
	Hypertension	1.73 (0.98–3.06)	0.058	1.71 (0.88–3.31)	0.114
	Hyperlipidemia	1.74 (0.96–3.12)	0.070	1.38 (0.64–2.94)	0.411
	Previous stroke	1.95 (1.06–3.58)	0.032	1.62 (0.74–3.51)	0.225
	NIHSS at admission	1.07 (1.03–1.12)	0.002	1.06 (1.01–1.12)	0.013
	Any ICH	2.49 (1.46–4.27)	0.001	2.98 (1.62–5.47)	<0.001
	Respiratory failure	10.39 (2.37–45.60)	0.002	8.12 (1.74–37.82)	0.008
**Non-DM group (*n* = 346)**					
30 days mortality	GAR				
	Q1	Reference		Reference	
	Q2	1.87 (0.53–6.63)	0.330	1.43 (0.332–6.17)	0.630
	Q3	4.19 (1.32–13.26)	0.015	3.27 (0.84–12.63)	0.086
	Q4	9.27 (3.01–28.70)	<0.001	4.28 (1.13–16.22)	0.032
	Current smoking	0.49 (0.244–0.99)	0.046	0.73 (0.29–1.78)	0.488
	NIHSS at admission	1.13 (1.07–1.20)	<0.001	1.15 (1.06–1.24)	<0.001
	Any ICH	9.29 (4.01–21.51)	<0.001	10.53 (3.83–28.97)	<0.001
	Respiratory failure	13.45 (5.93–30.53)	<0.001	19.27 (6.49–57.12)	<0.001
3-month Poor outcome (mRS > 2)	GAR				
	Q1	Reference		Reference	
	Q2	1.16 (0.63–2.14)	0.643	0.98 (0.51–1.91)	0.956
	Q3	1.82 (0.99–3.33)	0.051	1.37 (0.71–2.64)	0.346
	Q4	4.86 (2.49–9.49)	<0.001	3.39 (1.66–6.96)	0.001
	Age	1.03 (1.01–1.05)	0.003	1.02 (1.00–1.04)	0.045
	Coronary heart disease	2.03 (1.14–3.62)	0.017	1.09 (0.56–2.17)	0.789
	Atrial fibrillation	2.63 (1.50–4.62)	0.001	1.53 (0.79–2.95)	0.201
	NIHSS at admission	1.14 (1.09–1.19)	<0.001	1.12 (1.07–1.18)	<0.001
	Any ICH	2.61 (1.68–4.06)	<0.001	2.30 (1.41–3.76)	0.001

ICH, intracranial hemorrhage; mRS, modified Rankin score. GAR, glucose-to-glycated hemoglobin ratio; GAR Q1, first GAR quartile; GAR Q2, second GAR quartile; GAR Q3, third GAR quartile; GAR Q4, fourth GAR quartile.

### Binary logistic regression analysis of stress-induced hyperglycemia and clinical outcomes

The results noted, by comparing the lowest quartile, the fourth quartile of GAR in the non-DM group was independently associated with poor prognosis on 3-month (OR 3.39, 95% CI 1.66–6.96, *p* = 0.001) after adjusting risk factors. In addition, by comparing to the lowest quartile in both DM and non-DM group, GAR in the fourth quartile were found to be independently linked with 30 days mortality (OR 4.36, 95% CI 1.25–15.25, *p* = 0.021) and (OR 4.28, 95% CI 1.13–16.22, *p* = 0.032) after adjusting the confounders. Additionally, intracranial hemorrhage was an independent indicator for 30 days mortality and poor prognosis at 3-month both in DM and non-DM group. NIHSS at admission independently associated with poor prognosis at 3-month in two groups while not associated with 30 days mortality in DM group. Respiratory failure during hospitalization had a relation to 30 days mortality in the two groups and was associated with poor prognosis at 3-month in the DM group (Table 1).

## Discussion

The present study has demonstrated that the impact of stress hyperglycemia on 3-month prognosis in AIS patients after EVT was influenced by premorbid diabetic status. Although overall outcomes of DM patients appear to be worse than non-DM patients, those without history of diabetes showed significantly worse outcomes at 3 months after developing stress-induced hyperglycemia.

Recent clinical studies have defined stress hyperglycemia using the fasting blood glucose within 24 h (Merlino et al., 2021a,b; Mi et al., 2022; Ngiam et al., 2022; Tao et al., 2022). In fact, an animal study revealed a leak level of glucose after 24 h of focal cerebral ischemia and this practically normalized on the third day (Harada et al., 2009). In addition, a recent study of rats with global cerebral ischemia demonstrated decreases in hepatic expression of mRNA for pyruvate carboxylase (50%), PEPCK (56%), and G6Pase (80%) at 24 h after ischemia, which resulted in profoundly reduced gluconeogenesis in the liver (Sá-Nakanishi et al., 2020). A clinical study showed that persistent hyperglycemia in 24–48 h after stroke onset was not associated with worse functional prognosis at 3 months, regardless of patients’ diabetic history (Ntaios et al., 2011).

Glycated hemoglobin ratio has been considered a novel marker of poor outcomes in patients with ischemic stroke after IVT and MT (Chen et al., 2019; Tsivgoulis et al., 2019; Suissa et al., 2020; Merlino et al., 2021a; Shen et al., 2021; Cannarsa et al., 2022). This study compared GAR and poor prognosis in patients treated by EVT, based on their history of DM. DM group had more patients with poor prognosis and higher GAR. Patients with DM showed more severe stress-related hyperglycemia and worse prognosis at 3-month than non-DM group. Each group was divided into four quartiles to detect associations between GAR and clinical outcomes and any confounding factors for the above findings.

The results initially noted that elevated GAR was associated with a 3-month poor prognosis and mortality at 30 days in DM and non-DM groups. However, elevated GAR did not directly indicate a 3-month poor prognosis in DM group after adjusting several concomitant variables by logistics regression. This, overall, illustrated the different responses from DM vs. non-DM patients to stress-induced hyperglycemia associated with LVO stroke. Furthermore, types of stroke appeared to be different in non-DM patients (less LAA) with the highest GAR quartile, suggesting an alternative etiology that needs further investigation.

Controlling glucose levels to the range of 140–180 mg/dl (7.7–10 mmol/L) after AIS is advised by the American Heart Association/American Stroke Association and the European Stroke Organization [European Stroke Organisation (Eso) Executive Committee, 2008; Rabinstein et al., 2019]. Chinese guidelines for diagnosis and treatment of acute ischemic stroke 2018 suggest maintaining blood glucose from 7.8 to 10 mmol/l (Zhong et al., 2018). Many randomized and open observational studies, however, refute these suggestions. The 24-h post-ischemic intense insulin injection for AIS patients has not shown any advantage in the randomized UK Glucose Insulin in Stroke Trial (GIST-UK) (Gray et al., 2007). There has been no discernible positive functional effect from intensive insulin therapy keeping blood glucose within the range of 4–7.5 mmol/L (Bellolio et al., 2014; Ntaios et al., 2014; Yamada et al., 2017; Johnston et al., 2019; Kamal et al., 2020; Hollist et al., 2021).

However, patients without history of DM who develop stress-induced hyperglycemia appear to benefit greatly from the glycemic control treatment. For instance, critical patients without pre-existing DM have shown better clinical outcomes with blood glucose management than those with history DM (Krinsley, 2006). Also, differences between patients with and without DM have been found in time-weighted average glucose levels, the occurrence of hypoglycemia, and variability of blood glucose levels, ultimately impacting their clinical outcomes; patients with DM have a more blunted response to hypo- and hyperglycemia and changes in blood glucose levels (Fong et al., 2022). Similar findings seem to be present with stress-induced hyperglycemia associated with AIS, which emphasizes the importance of previous glycemic status. This may guide future studies on therapeutic options for patients with AIS.

The different clinical outcomes from stress-induced hyperglycemia in patients with and without diabetes can also be attributed to pathologic changes in response to chronic hyperglycemia (Ma et al., 2020; Ferrari et al., 2022). High serum blood glucose can notably influence cerebral vascular structure and body metabolism (Ergul et al., 2012). The hyperglycemic state may promote high levels of plasma plasminogen activator inhibitor-1 (PAI-1), which can be responsible for hypercoagulative status in ischemic stroke (Tjärnlund-Wolf et al., 2012; Chen et al., 2017). Advanced glycation end products (AGEs) and their receptors are found in patients with DM and they increase the production of reactive oxygen species (ROS), resulting in the activation of the nuclear transcription factor (NF-κB) and transcription of pro-inflammatory genes, IL-1, IL-6, and TNF-α (Indyk et al., 2021). AGEs are associated with diabetic complications from atherosclerosis and involved in the aging of blood vessels and numerous sequential damages (Toprak and Yigitaslan, 2019; Ahmad et al., 2020). More studies are necessary to determine if stress hyperglycemia is responsible for worse outcomes with or without EVT or if this is a marker of sympathetic/metabolic derangement. This question may ultimately help target the root cause of the poor outcomes.

The mechanism of stress-induced hyperglycemia after stroke appears to be due to consequences of increased hepatic gluconeogenesis and reduced sensitivity to insulin (Liao et al., 2020). Upregulation of hepatic gluconeogenesis has been noted in hyperglycemic animal models after focal brain ischemia (Harada et al., 2012; Wang et al., 2014). Increased levels of glucagon, corticosterone, and norepinephrine in response to post-stroke hyperglycemia promotes hepatic gluconeogenesis (Chen et al., 2016). Acute brain ischemia has been found to enhance inflammatory pathways, the expression of hepatic TNF-α, and activities of intracellular NF-κB from catecholamine release, which ultimately results in hepatic insulin resistance (Wong et al., 2011; Belayev et al., 2020; Wu et al., 2021). Furthermore, levels of NF-κB and TNF-α in diabetic patients are different from those in non-diabetic patients. Chronic hyperglycemia and the changes of stress hormones after AIS could lead to higher GAR and worse prognosis in diabetic patients in addition to the damages caused by chronic metabolism derangement and vascular damage.

Drugs for glycemic control may have neuroprotective effects. Acarbose can exert neuroprotective effects by preventing mitochondrial and lysosomal dysfunction. It also modifies gene expression related to inflammation, cell survival, and regeneration by suppressing P53 protein which performs as a signaling point for the convergence of necrosis and apoptosis in cerebral ischemia (Tseng, 2020; Cozene et al., 2021; Das et al., 2022). Meformin improves neurological functions of acute stroke patients with DM II and the oxidative stress levels (Zhao et al., 2019). Selective agonists of glucagon-like peptide-1 receptors (GLP-1Ras) have shown neuroprotective effects by exerting anti-apoptotic and anti-edema actions and promoting microcirculation and the brain-blood-barrier integrity (Zhu et al., 2016; Shan et al., 2019; Zhao et al., 2020). Clinical studies have confirmed that the beneficial impact of GLP-1 receptor agonists in treating hyperglycemia of AIS patient (Daly et al., 2013).

There were a few limitations in this study. First, this was a retrospective and single-center study. Unforeseen errors may have occurred because of the nature of the study design and discrepancies in practicing medicine among different geographic regions. Secondly, studies in the past have shown that stress-induced hyperglycemia peaks in 24 h and returns to normal on the third day (Harada et al., 2009); fasting glucose levels were measured within 24 h of the onset of symptom in this study. Finally, glycemic variability (GV), another important component of abnormal glucose levels associated with worse functional outcomes in AIS patients with DM (Kim et al., 2017), was not included in this study.

## Conclusion

History of DM seems to influence outcomes of patients with AIS treated by EVT. Contrary to the non-DM group, stress-induced hyperglycemia is not an independent predictor of poor outcome for patients with DM. This may be due to adaptive responses to chronically high serum glucose levels and glucose metabolism. Highest quartile of non-DM group stress hyperglycemic responders appear to behave differently than the other groups from cerebrovascular etiology perspective. These findings may have roles in future study about neuroprotection and improving outcomes of advanced stress hyperglycemic responders.

## Data availability statement

The datasets presented in this study can be found in online repositories. The names of the repository/repositories and accession number(s) can be found in the article/supplementary material.

## Ethics statement

Each study participant signed a consent form before undergoing EVT. A consent waiver from the Ethics Committee of Beijing Luhe Hospital was received because of the retrospective study design.

## Author contributions

HD: conceptualization, methodology, software, formal analysis, and writing—original draft. HY: writing and editing original draft. GR: writing and review. FC: investigation and software. YW: data curation and methodology. JL: data curation and investigation. LC: resources and supervision. YT: software and validation. ZC: visualization. XG and YD: conceptualization, funding acquisition, resources, supervision, and writing—review and editing. All authors contributed to the article and approved the submitted version.
